# The Art of Embalming

**Published:** 1888-09-08

**Authors:** 


### The Art of Embalming.

An old art and a new one; an honour once reserved for the great and honoured, and now chiefly recommended for the supposed victims of crime; a thing which those who cling to life crave for, and those who most revere death shrink from; a practice whose very name brings together the extremes of old and new civilisation, as popular in the America of to-day as in the Egypt of the Pharaohs—such is the art of embalming. The method employed now is very different from that of the Egyptian priest-physicians, but the idea is the same in most cases—the fond, pathetic longing of the living to retain the semblance of life in the beloved dead after the reality has departed.

The old method of embalming consisted in removing the nternal organs from the body, and filling up the vacant space with spices and aromatic herbs of various sorts. This style, though more romantic to the ear, has been abandoned, and various chemical substances, among them that terrible poison, corrosive sublimate, are now preferred. William Hunter, the anatomist, is generally credited with having invented the new fashion of embalming by injection, but the credit really belongs to a famous Dutch anatomist, Frederick Ruysch, who was professor of anatomy at Amsterdam between the years 1665 and 1717. The means he employed are unknown, for he died without confiding his secret to either friend or pupil, but the specimens he produced of embalmed bodies formed one of the curiosities of his age. Among the many visitors who came to his studio, or dissecting-room, was the royal tourist and knowledge-seeker, Peter the Great, who was so struck with the specimens put before him, that he kissed the lips of a child whose body Ruysch had preserved, unconscious for the moment that it was dead. The eccentric Czar purchased a cabinet of Ruysch's specimens and carried them off to St. Petersburg.

Ruysch's method being thus lost to posterity, the credit of introducing the new method of embalming remains to William Hunter. His principle was the same as the Dutchman's, namely, forcing a preservative fluid through the blood-vessels of the body, the initial opening being made in the femoral artery. The fluid he chose was a solution of oil of turpentine, Venice turpentine, oil of lavender, oil of camomile, and vermilion. When the body had been sufficiently saturated with this mixture, it was placed in a coffin on a dry bed of plaster of Paris, intended to draw out all moisture from it, and there it was left for four years, by which time the desiccation was, as a rule, complete. Several specimens of Hunter's work are preserved in the Hunterian Museum in the University of Glasgow, and they are remarkable for the skill with which the expression, as well as the outline, of the features has been preserved. Hunter's method was followed for a long time, and has not yet been fundamentally superseded, though new substances have been chosen for the injecting fluid.

M. Bondet, a Frenchman, introduced the use of corrosive sublimate, while after him another of the same nation, Franchina, employed arsenic dissolved in spirit of wine in the proportion of one part to ten. So common did the practice of embalming bodies with these arsenical solutions become in France, that the government of Louis Phillipe issued an order in 1846 prohibiting the sale of arsenic and all compositions containing it, which were to be used for the soaking of grain, the destruction of insects, and the embalming of bodies.

In our own day, embalming is practised among Americans more than any other nation. The method employed is founded on that of M. Lucquet, a contemporary of Franchina's, who chose as his preservative fluid a solution of chloride of zinc. The usual intention is merely to preserve the body an unusually long time before interment, as when it is desired to take it to another country from that where death took place. Thus there are few demands made on the embalmer's skill, and though it is still a fashion, because a sign of wealth, with our transatlantic cousins, it is chiefly the demands of science that preserve any knowledge of the art of embalming.

